# *Tetragonia tetragonioides* Relieves Depressive-Like Behavior through the Restoration of Glial Loss in the Prefrontal Cortex

**DOI:** 10.1155/2021/8888841

**Published:** 2021-02-12

**Authors:** Yujin Choi, Yunna Kim, Hwa-Young Lee, Seung-Hun Cho

**Affiliations:** ^1^Clinical Medicine Division, Korea Institute of Oriental Medicine, Daejeon 34054, Republic of Korea; ^2^Department of Clinical Korean Medicine, Graduate School, Kyung Hee University, Seoul 02447, Republic of Korea; ^3^Department of Neuropsychiatry, College of Korean Medicine, Kyung Hee University, Seoul 02447, Republic of Korea; ^4^Research Group of Neuroscience, East-West Medical Research Institute, WHO Collaborating Center, Kyung Hee University, Seoul 02447, Republic of Korea

## Abstract

*Tetragonia tetragonioides*, which is a halophyte and grows widely in Asian-Pacific regions, has been used for the treatment of digestive disorders in traditional oriental medicine. This study examined the potential antidepressant effect of *Tetragonia tetragonioides* in an astroglial degeneration model of depression, which was established based on the postmortem study of depressive patients' brain presenting diminished astrocytes in the prefrontal cortex. C57BL/6 male mice were exposed to glial ablation in the prefrontal cortex by the administration of the gliotoxin, L-alpha-aminoadipic acid (L-AAA) to induce depression. *Tetragonia tetragonioides* at doses of 100 mg/kg and 300 mg/kg, imipramine at a dose of 15 mg/kg, and distilled water were orally administrated to mice for 18 days. Behavioral tests including the open field test (OFT), sucrose preference test (SPT), forced swimming test (FST), and tail suspension test (TST) were carried out after 2 days of L-AAA injection. The expression levels of GFAP and NeuN in the prefrontal cortex were determined by immunohistochemistry. Mice subjected to glial ablation in the prefrontal cortex displayed decreased sucrose consumption in SPT and increased immobility time in FST and TST. Treatment with imipramine and *Tetragonia tetragonioides* remarkably ameliorated the behavioral despair induced by L-AAA. In addition, immunohistochemistry analysis showed that treatment with *Tetragonia tetragonioides* significantly restored the glial loss as indicated by the elevated GFAP expression level. These findings suggest that *Tetragonia tetragonioides* exerts an antidepressant effect through the restoration of glial loss under conditions of depression and can be a candidate for an antidepressant agent.

## 1. Introduction

A depressive disorder is identified as characteristic symptoms of despair, anhedonia, loss of appetite or sleep disturbance, decreased energy and concentration, and feeling of guilt and worthlessness [1–5]. According to the epidemiological survey of mental disorders in Korea in 2011, the lifetime prevalence of major depressive disorder was reported to be 6.7% [6]. As per the report on death and causes of death in Korea, the suicide rate was 24.3 per 100,000 persons in 2017 [7]. Needs for effective and immediate treatments for depression with fewer adverse events are gradually increasing, and various local herbs have been tested for this purpose [8–10].

In the last few years, pathology of glial cells has been studied with emphasis to understanding the mechanisms behind brain disorders [11]. Especially, decreased density of glial cells in the prefrontal and the cingulate area was consistently reported in depressed patients [12]. Abnormalities in glial cells, especially astrocytes, play an important role in mediating major depressive disorders. The previous review summarized that the expression of the glial fibrillary acidic protein (GFAP) and representative astrocyte related proteins was significantly decreased in subjects with major depressive disorders [13, 14]. In animal studies, chronic and acute stress induced depressive-like symptoms in behavior and alterations in astrocytes in the brain [15].

Immense efforts have been made to generate the valid and insightful model of depression, and the chronic unpredictable stress model is one of the well-documented animal models for depression [16]. Based on the postmortem research studies reporting a loss of glia in prefrontal and the cingulate area from depressed patients [17], Banasr and Duman proved that glial ablation in the prefrontal cortex induced depressive-like behaviors [18]. The authors provoked the astrocytic degeneration by infusing L-alpha-aminoadipic acid (L-AAA), a gliotoxin specific for astrocytes [19], into prefrontal cortex. Infusion of L-AAA only led to the loss of glia and not neurons. Infusions of ibotenic acid, which is toxic for neurons, did not induce the depressive-like behaviors.

*Tetragonia tetragonioides* (Pall.) Kuntze (TTK), commonly called as New Zealand spinach, belongs to the family of Aizoaceae, and widely grows in Korea, Japan, southeast China, and New Zealand [20, 21]. In Korea, TTK grows abundantly on the Jeju Island [22]. In traditional oriental medicine, TTK has been documented to impart the protective effect in conditions of digestive troubles, and the antiulcerogenic activity was also reported [23]. Extract of TTK includes various antioxidative compounds [24], and TTK is reported to have antioxidant and anti-inflammatory effects [25]. 70% ethanol extract of TTK demonstrated potential to treat menopause-associated symptoms and metabolic disturbances [26]. Moreover, TTK extract ameliorated the depressive-like symptoms and regulated the serotonin level in ovarietomized rats [27]. The antidepressant effect of TTK in the depressive models has not been tested, and previous studies indicated the possibility that TTK may be effective on treating depressive disorders.

To address this topic, a depression model of astroglial degeneration was used to investigate the ameliorative effects of TTK on depressive symptoms. Furthermore, we examined the possible mechanism behind the antidepressant-like effects of TTK by examining the expression of the GFAP in the prefrontal region in mice brain.

## 2. Materials and Methods

### 2.1. Animals

Fifty male C57Bl/6 mice, aged seven weeks, were purchased from Orient Bio Inc., Korea. The mice were housed in acrylic cages (20 cm × 27 cm × 12 cm) under standard experimental conditions at constant temperature (22 ± 2°C), humidity (60 ± 10%), and light (12-hour light and dark cycle, with lights off at 6 pm). Animals were allowed free access to food and water and had a period of acclimation before the start of each experiment. The Kyung Hee University Medical Center Institutional Animal Care and Use Committee approved all the procedures (KHMC-IACUC 16-033). All efforts were made to minimize animal suffering during the progression of the experiments.

### 2.2. Drugs and Reagents

TTK was obtained from Jeju, Korea. The whole dried TTK (600 g) was boiled twice with 30% ethanol, and the extract was filtered through a filter paper. The filtrate was concentrated using a rotary evaporator and lyophilized to yield a powder (91 g). The powder was stored at 4°C until use. Mouse monoclonal antibodies against *β*-actin and GFAP were obtained from Santa Cruz Biotechnology (CA, USA). Neuronal-specific nuclear protein (NeuN) was purchased from Millipore Inc. (Bedford, MA, USA). Horseradish peroxidase-conjugated antimouse secondary antibody was purchased from Pierce Biotechnology (Rockford, IL, USA). Acetonitrile and methanol were purchased from Honeywell Burdick and Jackson (Morristown, NJ, USA) and were of HPLC grade. Analytical-grade formic acid (99% purity) was obtained from Sigma-Aldrich (St. Louis, MO, USA). Deionized water (>18 mΩ) was obtained via a pure water purification system (Human Co., Korea).

### 2.3. LC-QTOF-MS Analysis

Isoferulic acid and 4-hydroxybenzoic acid were selected for reference standards based on previous studies. Approximately 50 mg of TTK powder was shaken with 1 mL of methanol by a vortex mixer for 30 seconds. The supernatants were filtered through a 0.2 *μ*m polytetrafluoroethylene syringe filter (Thermo Scientific). Finally, the filtrate was transferred to a LC sample vial before use.

The liquid chromatography-mass spectrometry system consisted of a Thermo Scientific Vanquish UHPLC system (Thermo Fisher Scientific, Sunnyvale, CA, USA) with an Acclaim RSLC 120 C18 (2.1 mm × 100 mm, 1.7 *μ*m; Agilent Technology, Ca, USA) and a TripleTOF 5600+ mass spectrometer system (TripleTOF MS; QTOF, Sciex, Foster City, CA, USA). The QTOF MS, equipped with a DuoSprayTM ion source, was used to complete the high-resolution experiment. The LC gradient used a mobile phase A containing 0.05% formic acid and 2.5 mM ammonium formate in water and a mobile phase B of acetonitrile. The flow rate was kept constant at 0.4 mL/min, and the injection volume was 1 *μ*L. The gradient elution system began at 5% B for 0.8 min, 5–20% B from 0.8 to 2.5 min, 20–32% B from 2.5 to 5.5 min, 32–38% from 5.5 to 8, 38–45% B for 2 min, 45–60% B for 2 min, 60-95% B for 4 min, then increased to 100% B at 20.0 min, held at 100% B for 3 min, and then returned to the initial conditions for reequilibration.

Mass data acquisition was performed with a TripleTOF 5600+ in the negative ion mode using the following parameters: source temperature was set at 450°C with a curtain gas flow of 25 L/min (GS1 and GS2 both 50), the ion spray voltage was set at −4500 V, declustering potential was 30 V, and the collision energy was 10 V. High-purity nitrogen gas was used for the nebulizer/DuoSprayTM and curtain gases. The QTOF and information-dependent acquisition (IDA) scan were operated with a mass range of 50–1500 m/z. Precursor and product ion calibrations were performed in both high sensitivity and high-resolution modes using a calibrant delivery system prior to analysis. Data acquisition and processing were carried out using Analyst TF 1.7, PeakVeiw2.2, and MasterView (Sciex, Foster City, CA, USA).

### 2.4. Cannula Implantation

Mice were anesthetized using intraperitoneal injection of 100 mg/kg ketamine + 10 mg/kg xylazine, and guide cannula (RWD Life Science Co., Ltd., Shenzhen, China) were bilaterally implanted into the prefrontal cortex region of mice brain using a stereotaxic apparatus (Vernier Stereotaxic Instrument, Leica Biosystems, Nussloch, Germany) by the following coordinates: 1.7 mm anteroposterior, ±0.25 mm dorsolateral, and depth –2.5 mm from the bregma [28]. After seven days of recovery, we infused L-AAA (100 *µ*g/*µ*l; Sigma) bilaterally using injection cannula and a microdriven pump (Pump 11 Elite Nanomite, Harvard Apparatus, Holliston, MA, USA). We administered infusion once daily for 2 days at a rate of 0.1 *µ*l/min for 6 minutes.

### 2.5. Drug Administration

**Figure 1:**
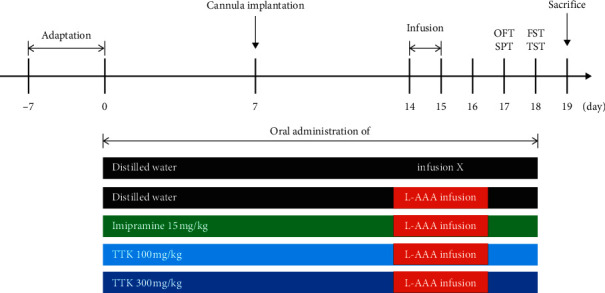
Experimental schedule. 50 mice were randomly divided into five groups (*n* = 10 for each group): control (sham surgery + distilled water), negative control (L-AAA injection + distilled water), positive control (L-AAA injection + imipramine 15 mg/kg), low-dose (L-AAA injection + TTK 100 mg/kg), and high dose (L-AAA injection + TTK 300 mg/kg). After 2 weeks of administration of drugs or distilled water, cannula implantation and infusion of L-AAA were performed. Subsequently, behavior tests including the open field test (OFT), tail suspension test (TST), forced swimming test (FST), and sucrose preference test (SPT) were carried out.

Mice were randomly assigned into five groups: control group with distilled water (DW), negative control group with L-AAA infusion and DW, positive control group with L-AAA infusion and 15 mg/kg imipramine, and two experimental groups with L-AAA infusion and 100 mg/kg or 300 mg/kg TTK. After the adaptation period, oral administration was continued until the animals were sacrificed. Experimental procedure that followed the time schedule is shown in Figure 1.

### 2.6. Behavioral Test

The open field test (OFT) was carried out to assess the locomotor activity [29]. Briefly, mice were placed in the center of a white, acrylic, plastic square box (50 cm × 50 cm) and allowed to freely explore the apparatus for 10 min. The total distance travelled, recorded by video camera, was evaluated using a computer-aided control system (SMART 3.0, Panlab Harvard Apparatus).

The sucrose preference test (SPT), tail suspension test (TST), and forced swimming test (FST) were conducted to measure the depressive behavior of the mice. In SPT, mice were habituated for 48 hours to 1% sucrose, and following a 4-hour of deprivation period, preference for sucrose or water was determined for 1 hour. The bottles of sucrose and water were identical [30]. For TST, mice were suspended 50 cm above the floor by adhesive tape placed 1 cm from the tip of the tail for 6 min. Immobility time was defined after the 2 min mark, as the duration of time the animal was hung passively and completely motionless during the remaining 4-minute period [31]. In FST, mice were placed in an inescapable open cylindrical container (diameter 20 cm, height 35 cm), with 15 cm of water maintained at 25°C for a total of 6 min. Immobility time was defined as the time the mouse ceased struggling and floated motionless during the last 4 min of the 6-minute test, following an initial 2 min of activity [32].

### 2.7. Immunohistochemistry

Mice were quickly anesthetized with diethyl ether and then perfused with phosphate-buffered saline (PBS), followed by 4% paraformaldehyde solution. The brains were fixed in 4% paraformaldehyde for 24 h, followed by PBS containing 20% sucrose for 24 h. Typically, 10 *μ*m thick coronal sections of each brain were embedded in optimal cutting temperature (OCT) compound and cut with a cryostat. After washing in PBS, the sections were incubated for 1 h at room temperature with 1% normal horse serum in PBS and incubated overnight at 4°C with the primary antibody against the GFAP and NeuN at 1 : 500 dilutions in PBS, containing 2.5% normal horse serum. After washing in PBS, sections were incubated for 1 h at room temperature with the biotinylated secondary antibody (1 : 50) in PBS containing 2% normal horse serum and subsequently incubated with ABC reagents (Vector Laboratories, CA, USA) in PBS. After washing again in PBS, the sections were incubated in 3,3'-diaminobenzidine tetrahydrochloride (DAB; Dako, CA) and mounted in permount mounting medium (Fisher Scientific International, USA). GFAP and NeuN-positive cells were detected using the Olympus BX51 microscope (Olympus, Tokyo, Japan)

### 2.8. Enzyme-Linked Immunosorbent Assay (ELISA)

Prefrontal cortex of some mice were dissected and stored at −80°C for ELISA. Tissue lysates were quantified according to the mouse ELISA kit (ThermoFisher, Waltham, Massachusetts, U.S, CAS: BMS607-3) manufacturer's user guide. Protein level of tumor necrosis factor-alpha (TNF-*α*) was measured at an absorbance of 450 nm (pg/ml) through a microplate reader.

### 2.9. Statistical Analysis

Statistical differences of the mean were examined using one-way analysis of variance (ANOVA), followed by Dunnett's test for intergroup comparisons. For all the analyses, a *P* value less than 0.05 was considered statistically significant. The analysis was conducted using SPSS 22.0 (IBM Inc., Armonk, NY, USA).

## 3. Results

### 3.1. Chromatogram of TTK Extract and Reference Standards

**Figure 2:**
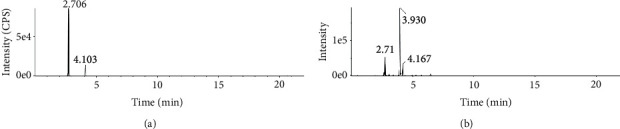
Extracted ion chromatogram (XICs) of 4-hydroxybenzoic acid and isoferulic acid from (a) reference standards and (b) TTK 30% ethanol extract. The peak at 2.71 is expected to 4-hydroxybenzoic acid, and the peak at 4.1 is expected to isoferulic acid. In Figure 2(b), the peak at 3.93 is expected to ferulic acid.

Extracted ion chromatogram of 4-hydroxybenzoic acid and isoferulic acid from reference standards and TTK powder is shown in Figure 2. Peak of 4-hydroxybenzoic acid is seen at 2.71 min and that of isoferulic acid is seen at 4.10 min. In TTK power, 4-hydroxybenzoic acid (RT: 2.71) and isoferulic acid (RT: 4.17) was confirmed to exist from the MS/MS spectrum, and the peak at 3.93 is expected to ferulic acid.

### 3.2. Effect of TTK on the Behavior Tests in L-AAA Injected Mice

**Figure 3:**
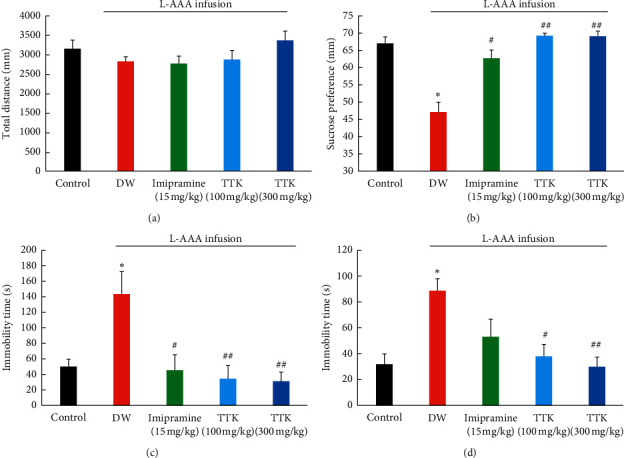
Effect of TTK in the behavior test. (a) Total distance in the open field test (OFT), (b) sucrose preference in the sucrose preference test (SPT), (c) immobility time in the forced swimming test (FST), and (d) immobility time in the tail suspension test (TST). ^∗^*P*<0.05 as compared with the control group; ^#^*P*<0.05 as compared with L−AAA+DW group; ^##^*P*<0.01 as compared with L−AAA+DW group.

The locomotor activity of mice was measured in an open field test (Figure 3(a)). There was no significant difference observed among the groups with respect to the total distance moved (*F* (4,42) = 1.627, *P* = 0.185). Thus, it was apparent that L-AAA infusion, administration of imipramine, and TTK did not affect the locomotor activity of mice.

The depressive-like behaviors of mice were measured by the sucrose preference test, tail suspension test, and forced swimming test. The result of the sucrose preference test is presented in Figure 3(b). The percentage of sucrose preference was significantly reduced in the L-AAA + DW group, compared with the control group (*P*=0.003). TTK at doses of 100 mg/kg (*P*=0.003) and 300 mg/kg (*P*=0.002) and imipramine at a dose of 15 mg/kg (*P*=0.016) showed a significant increase in sucrose preference compared with the L-AAA + DW group.

Figures 3(c) and 3(d) show the effect of L-AAA infusion on the immobility times of mice in FST and TST, respectively. The L-AAA + DW group displayed significantly increased immobility time compared to the control group in FST (*P*=0.037) and TST (*P*=0.004). TTK at doses of 100 mg/kg (*P*=0.008) and 300 mg/kg (*P*=0.006) and imipramine at a dose of 15 mg/kg (*P*=0.016) showed a significant decrease in immobility times compared with the L-AAA + DW group in FST (Figure 3(c)). TTK at doses of 100 mg/kg (*P*=0.018) and 300 mg/kg (*P*=0.003) significantly decreased the duration of immobility time, whereas imipramine at a dose of 15 mg/kg (*P*=0.177) did not show a significant decrease compared with the L-AAA + DW group in TST (Figure 3(d)).

### 3.3. Effect of TTK on GFAP and NeuN Expression in L-AAA Injected Mice

**Figure 4:**
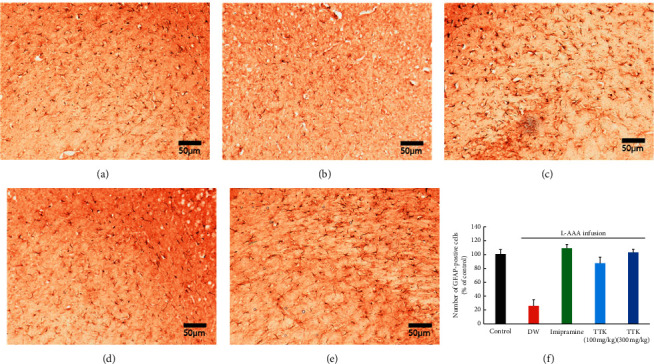
Effect of TTK on expression of GFAP-positive cells in the prefrontal cortex of mice treated with L-AAA as determined by immunohistochemistry. Representative coronal sections of the mice brain in (a) sham surgery + distilled water, (b) L-AAA + distilled water, (c) L-AAA + imipramine (15 mg/kg), (d) L-AAA + TTK (100 mg/kg), and (e) L-AAA + TTK 300 mg/kg.

The outcomes of immunoreactivity of GFAP in the prefrontal region are demonstrated in Figure 4. Slides from control mice displayed moderate GFAP immunoreactivity in the prefrontal region (Figure 4(a)), whereas slides from the L-AAA + DW group revealed lowered immunoreactivity patterns (Figure 4(b)). Notably, slides from mice treated with TTK and imipramine also displayed a moderate level of GFAP immunoreactivity in the prefrontal region (Figures 4(c)–4(e)), similar to the slides prepared from the control mice.

**Figure 5:**
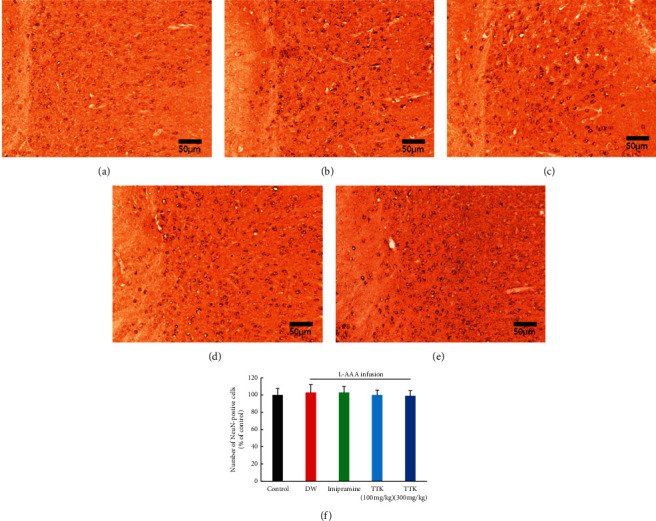
Effect of TTK on expression of NeuN-positive cells in the prefrontal cortex of mice treated with L-AAA as determined by immunohistochemistry. Representative coronal sections of the mice brain in (a) sham surgery + distilled water, (b) L-AAA + distilled water, (c) L-AAA + imipramine (15 mg/kg), (d) L-AAA + TTK (100 mg/kg), and (e) L-AAA + TTK 300 mg/kg.

The outcomes of immunoreactivity of NeuN in the prefrontal region are shown in Figure 5. Slides from all mice displayed moderate NeuN immunoreactivity in the prefrontal region. There was no significant difference observed in the NeuN expression among the groups.

### 3.4. Effect of TTK on TNF-*α* in L-AAA Injected Mice

**Figure 6:**
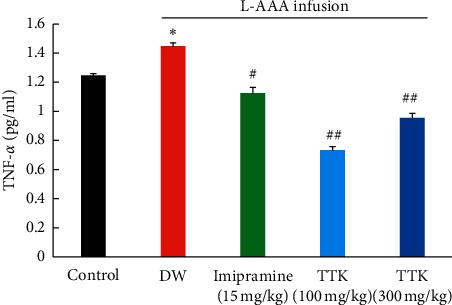
Effect of TTK on the level of TNF-*α* in the prefrontal cortex of mice treated with L-AAA. ^∗^*P*<0.05 as compared with the control group; ^#^*P*<0.05 as compared with L−AAA+DW group; ^##^*P*<0.01 as compared with L−AAA+DW group.

The protein level of TNF-*α* determined by ELISA is shown in Figure 6. The protein level of TNF-*α* in the prefrontal cortex significantly increased in the L-AAA + DW group compared to the control group. In contrast, the protein level of TNF-*α* in the prefrontal cortex of mice treated with both low dose and high dose of TTK and imipramine were significantly lower than that of mice treated with DW.

## 4. Discussion

The major finding of our study is that TTK can alleviate the depressive symptoms induced by the astroglial degeneration model of depression in mice. Mice treated with TTK showed an increased activity and sucrose preference in behavior tests, compared with those treated with DW. TTK appeared to protect the decrease of GFAP expression induced by L-AAA injection in prefrontal cortex. Therefore, our results indicate that TTK may offer a potential alternative treatment for treating depressive disorders.

Increased immobility times in the TST and FST of L-AAA-infused mice reflect a state of despair observed in depression. Their decreased sucrose preference also reflects a loss of interest. In the present study, TTK imparted protection in sucrose preference in SPT and decreased immobile times in FST and TST without affecting mouse locomotor activity. Pathology of astrocytes contributes significantly to major depressive disorders [33, 34]. In the prefrontal cortex, astrocytes regulate glutamate levels, and blockade of astrocytic glutamate uptake is related to symptoms of anhedonia [35]. Also, low adenosine triphosphate abundance released by astrocyte in the brain modulates the symptoms of despair presented by increased immobility time [36]. Infusion of L-AAA in the prefrontal cortex of mice also reported to decrease the brain levels of induced glutamate and glutamine [37] and not only induce astrocytic degeneration.

These results from this study show that TTK attenuated the depressive-like symptoms produced by the astroglial degeneration model of depression. After the glial ablation model of depression was developed [18], the antidepressant-like effect of mGluR5 antagonist MTEP [38], prefrontal cortex deep brain stimulation [39], Y5 receptor antagonist Lu AA33810 [40], Harmine [41], ZL006 [42], and ginsenoside Rf [43] were tested. In a manner similar to a previous study, infusion of L-AAA induced depressive-like behavior, and administration of imipramine [38] reversed the depressive behavior and led to a decrease in the number of GFAP-positive cells. In this study, we demonstrated that L-AAA infusion induced a decreased expression of GFAP in the prefrontal region, which was ameliorated with the administration of TTK. TTK imparted a protective effect against the reduction of GFAP expression induced by L-AAA infusion. Numerous evidences demonstrate that a decrease in the number of glial cells in the prefrontal region reflects the pathology of depression, and it is encouraging to note that treatment with TTK recovered the glial ablation immediately.

The antidepressant effect of TTK is presumably related to inhibition of neuroinflammation. In this study, TTK reduced the level of TNF-*α*, which was similar to the previous report on the effect of TTK in estrogen-deficient rats [26]. TNF-*α* is one of the key proinflammatory cytokines, which is relevant to pathogenesis of depression [44, 45] through activating hypothalamopituitary-adrenocortical (HPA) axis. Administration of TNF-*α* was reported to induce depressive-like behavior in mice, which was diminished by treatment with typical antidepressants such as fluoxetine and imipramine [46]. The imipramine and fluoxetine also reduced the production of TNF-*α* both in human patients and in the animal model of depression [47–49]. Also, in the astroglial degeneration model of depression, L-AAA-injected mice showed elevated TNF-*α* [50]. The reversal of TNF-*α* by TTK suggests that TTK may elicit the antidepressant-like effect by participating in anti-inflammatory activity.

In the previous study, 1% and 2% TTK 70% ethanol extract decreased the immobility time in ovariectomized rats [27]. In this study, oral administration of 100 mg/kg and 300 mg/kg 30% ethanol extracts showed the antidepressant-like effect in the L-AAA induced depression model. Typically, 300 mg/kg TTK seems to be superior in relieving the depressive behavior compared to 100 mg/kg TTK in the behavior test, indicating a dose-dependent response. Ferulic acid is one of the active components of the extract from TTK [51]. Recently, the antidepressant-like effects of ferulic acid have been reported in various models of depression [41, 52–55]. As far as underlying mechanisms are considered, activity in response to neuroinflammation in the prefrontal cortex [53] and elevation of the neurotrophic factor in the prefrontal cortex and hippocampus [41] have been reported. In this study, TTK reversed depression-like behavior and protected the glial ablation. Ferulic acid may be the major component of TTK responsible for exerting an antidepressive effect, and quantitative comparison between the effect of single compound and extract of TTK needs further investigations.

There are several limitations to our study. First, the antidepressant-like effect of the new natural product was only tested in the astroglial degeneration model of depression. As the transitory glial ablation effect of L-AAA lasts for three days [18, 56], the glial ablation model of depression was used to test the acute effect of the drugs. In this study, after infusion of L-AAA, drug administration was performed twice before the conduction of behavior tests. We designed this study to test the preventive and acute effect of TTK. Further research studies are required to explore the long-term effect of TTK in another depression model such as the chronic unpredictable stress model. Second, the mechanism behind the antidepressive effect of TTK was not examined enough in this study. Considering the previous studies about the antioxidant and anti-inflammatory effects of TTK, the effect of TTK on another antidepressant mechanism in prefrontal cortex related to depression is also expected. Third, as TTK is the natural product, which contains various compounds, the active compound involved in exhibiting the antidepressive effect should be proved in the further research studies.

## 5. Conclusions

The present study demonstrates the potential of TTK in treating depressive symptoms and the alterations in the brain of the animal model of depression. TTK protected the mouse brain glial loss in the prefrontal cortex induced by a gliotoxin, L-AAA. It is suggested that TTK may be one of the potential candidates for treating depression. Further research studies are required to understand the effect of TTK on depressive disorders.

## Data Availability

The data used to support the findings of this study are available from the corresponding author upon request.
